# The Evolving Landscape of Reduced-Intensity Conditioning: Balancing Efficacy and Toxicity Across Hematologic Malignancies

**DOI:** 10.1007/s11899-026-00791-4

**Published:** 2026-10-29

**Authors:** Piyatida Chumnumsiriwath, Stefan O. Ciurea, Piyanuch Kongtim

**Affiliations:** 1 Hematopoietic Stem Cell Transplantation and Cellular Therapy Program, Division of Hematology/Oncology, Department of Medicine, University of California Irvine, Orange, CA 92868 USA; 2 Division of Hematology, Department of Medicine, Faculty of Medicine Naresuan Hospital, Naresuan University, Phitsanulok, Thailand

**Keywords:** Allogeneic stem cell transplantation, Reduced-intensity conditioning regimens (RIC), Myeloablative conditioning regimens (MAC), Graft-versus-tumor effects (GVT), Risk-adapted tools, Personalized conditioning regimen

## Abstract

**Purpose:**

Allogeneic hematopoietic stem cell transplantation (allo-HSCT) has evolved from traditional myeloablative conditioning toward reduced-intensity conditioning (RIC) and strategies using donor-derived immune effector cells. This review summarizes trends in RIC, proposes a practical classification of regimens, and underscores personalized risk stratification and disease-specific conditioning.

**Recent Findings:**

Fludarabine-based backbones combined with alkylating agents—melphalan (Flu-Mel), busulfan (Flu-Bu), or cyclophosphamide (Flu-Cy)—remain widely adopted due to favorable tolerability and antitumor activity. Large CIBMTR analyses indicate melphalan-based RIC confers superior long-term survival in older patients with myeloid malignancies, primarily through reduced relapse rates. The emergence of integrated prognostic tools such as the Hematopoietic Cell Transplant–Composite Risk (HCT-CR) score and the HCT Frailty Scale enhances pretransplant risk assessment and patient selection. Incorporation of measurable residual disease (MRD) testing and next-generation sequencing (NGS) chimerism refines conditioning intensity decisions and enables earlier relapse detection and intervention. While most prospective trials have focused on AML and MDS, accumulating evidence supports RIC efficacy in selected patients with ALL and various lymphomas. Collectively, data emphasize that optimal conditioning is disease-, risk-, and patient-specific, requiring tailored approaches.

**Summary:**

RIC has extended allo-HSCT to older, frailer patients, achieving outcomes comparable to myeloablative regimens despite relapse risk. Future work should prioritize prospective disease-specific trials comparing RIC regimens; integration of dynamic biomarkers (MRD, immune profiling, NGS chimerism) into adaptive conditioning; development of frailty-adjusted protocols; and evaluation of post-transplant cellular therapies and targeted maintenance to prevent relapse. These advances aim to personalize conditioning and post-transplant management to maximize survival and minimize toxicity.

## Introduction

Allogeneic hematopoietic stem cell transplantation (allo-HSCT) has long been recognized as a potential curative therapeutic approach for hematologic malignancies and selected non-malignant disorders. Conditioning regimens, also known as preparative regimens, are a fundamental component of allo-HSCT. Administered prior to stem cell infusion, they serve several critical purposes depending on the primary disease, including eradication of residual malignant cells, sufficient immunoablation of the recipient immune system to prevent graft rejection, and creation of marrow space to facilitate donor stem cell engraftment [1]. For decades, successful transplantation was limited primarily to younger, healthy patients who could tolerate the profound immunosuppression and severe toxicities associated with myeloablative conditioning (MAC) regimens. The traditional MAC approach, typically consisting of high dose chemotherapeutic agents with or without total body irradiation (TBI) [2], was designed to achieve complete tumor eradication through maximal cytoreduction. Unfortunately, this aggressive strategy came at significant cost of higher regimen-related mortality and could be applied mostly to younger patients. Development of reduced-intensity conditioning (RIC) regimens represented a fundamental paradigm shift in allo-HSCT. Beginning in the 1980s, pioneering work demonstrated that allo-HSCT did not necessarily require complete eradication of recipient hematopoiesis to achieve durable engraftment and therapeutic benefit [3]. Rather, the anti-tumor efficacy of allo-HSCT relies substantially on the recognition of residual tumor cells by donor immune cells through a graft-versus-tumor effect [4, 5]. This conceptual shift led to development of RIC regimens that facilitate donor cell engraftment with improved tolerability, thereby expanding allo-HSCT to older patients and those with significant comorbidities or reduced fitness [2]. Building upon this progressive understanding, a multitude of RIC regimens with different safety and toxicity profiles have since been pioneered and applied for wide spectrum of disease types and patient characteristics. This review aims to summarize the diverse patterns and evolving trends of these RIC regimens across various disease settings and their clinical implications.

### Definition and Classification of Reduced-Intensity Conditioning (RIC) Regimens

A formal definition of RIC was established by consensus at a 2006 workshop convened by the Center for International Blood and Marrow Transplant Research (CIBMTR), incorporating three defining features; reversible myelosuppression, low non-hematologic toxicity and induction of mixed chimerism [6, 7]. According to this definition, conditioning regimens including ≤ 500 cGy of TBI as a single fraction or ≤ 800 cGy in fractionated doses, busulfan ≤ 9 mg/kg, melphalan dose ≤ 140 mg/m^2^, or thiotepa dose ≤ 10 mg/kg are considered RIC regimens [7]. These thresholds distinguish RIC from both fully myeloablative conditioning (MAC) regimens and non-myeloablative (NMA) regimens, which rely even more on graft immune effects and use primarily with immunosuppressive conditioning. Though, the boundaries between these categories can be somewhat arbitrary and may vary considerably based on the combination of agents used, the order of administration, and individual patient factors such as age, comorbidities, and disease status at transplantation. In contemporary practice, RIC regimens most commonly incorporate fludarabine combined with an alkylating agent such as melphalan [8, 9], busulfan [10, 11], treosulfan [12], or cyclophosphamide [13], or fludarabine in combination with low dose TBI [14].

More recently, the European Society for Blood and Marrow Transplantation (EBMT) introduced the Transplant Conditioning Intensity (TCI) score to provide a more refined assessment of conditioning intensity beyond the conventional myeloablative conditioning (MAC) and reduced-intensity conditioning (RIC) classification. The TCI score assigns predefined intensity weights to individual conditioning components, including chemotherapy and total body irradiation (TBI), based on their cumulative administered doses (Table 1). The individual scores are summed to generate an overall TCI score, stratifies conditioning intensity into three groups- low (score 1–2), intermediate (score 2.5–3.5) and high (4–6). For example, intravenous busulfan at a cumulative dose of 12.8 mg/kg contributes 3 points, while fludarabine at 120 mg/m^2^ contributes 0.5 points, resulting in a total TCI score of 3.5 (intermediate intensity) [15]. The TCI score has been shown to predict non-relapse mortality (NRM) more accurately than the traditional MAC/RIC classification. Therefore, this scoring system may provide clinically meaningful guidance for tailoring conditioning intensity according to individual patient characteristics and risk factors [16, 17].

**Table 1:** Transplant Conditioning Intensity (TCI) scoring based on conditioning components and cumulative doses [15]

Conditioning component	Low-dose level (points)	Intermediate-dose level (points)	High-dose level (points)
TBI, fractionated (Gy)	≤ 5 (1)	6–8 (2)	≥ 9 (3)
Busulfan, IV (mg/kg)	≤ 6.4 (1)	9.6 (2)	12.8 (3)
Busulfan, oral (mg/kg)	≤ 8 (1)	12 (2)	16 (3)
Fludarabine (mg/m^2^)	≤ 160 (0.5)	≥ 180 (1)	—
Melphalan (mg/m^2^)	90–125 (1)	≥ 140 (2)	—
Cyclophosphamide (mg/kg)	< 80 (0.5)	≥ 90 (1)	—
Carmustine (mg/m^2^)	≤ 250 (0.5)	280–310 (1)	≥ 350 (1.5)
Thiotepa (mg/kg)	< 10 (0.5)	≥ 10 (1)	—
Cytarabine (g/m^2^)	< 6 (0.5)	≥ 6.5 (1)	—
Treosulfan (g/m^2^)	30 (1)	36 (2)	42 (3)
Clofarabine (mg/m^2^)	≤ 150 (0.5)	> 150 (1)	—
Etoposide (mg/kg)	≤ 50 (1)	> 50 (2)	—

Legend: *Gy* gray, *kg* kilogram, *mg* milligram, m^2^: square meter

### Evolution and Comparative Efficacy of Modern Reduced-Intensity Conditioning Regimens

The landscape of RIC regimens is diverse, broadly categorized by their primary chemotherapy components and the number of drugs involved, as well as their specific applications. Many contemporary RIC protocols are built upon fludarabine backbone, owing to its multifaceted benefits in allo-HSCT. Typically, fludarabine is administered intravenously at doses of 150–180 mg/m^2^ over 4–5 days, serving as primary lymphodepleting agent. This purine nucleotide analog has potent antileukemic and immunosuppressive properties, with its primary role in RIC being the effective depletion of endogenous lymphocytes and when using at RIC doses, fludarabine induces reversible myelosuppression [18, 19]. This selective T-cell depletion is essential for ensuring allograft acceptance, maximizing donor cell engraftment and immunologic recovery, while minimizing damage to non-hematologic organs [20]. Furthermore, fludarabine demonstrates synergistic activity when combined with alkylating agents and irradiation, primarily by inhibiting cellular DNA repair mechanisms following exposure to these DNA-damaging agents [21–23]. Consequently, it serves as an effective backbone for nearly all modern RIC protocols.

Among the most frequently utilized two-drug RIC combinations is fludarabine with busulfan (Flu-Bu or FB), particularly prevalent in the treatment of acute myeloid leukemia (AML) and myelodysplastic syndrome (MDS). Results from a prospective multicenter phase II study evaluating the efficacy of fludarabine and busulfan RIC regimen for elderly patients with AML in first complete remission showed promising outcomes with 42% disease-free survival (DFS) and NRM of only 15% at 2 years. In this study, total busulfan dose was 6.4 mg/kg [24]. Evidence suggests that lower-dose busulfan maintains therapeutic efficacy in RIC settings. Investigators from Dana-Farber Cancer Institute reported encouraging allo-HSCT outcomes in elderly AML patients using Flu-Bu RIC regimen with busulfan total dose of either 3.2 or 6.4 mg/kg. In this cohort, progression-free survival (PFS) was comparable (40% vs. 39% at 2 years, respectively) and 2-year NRM remained notably low at less than 10% in both dose levels [25].

The Fludarabine/Melphalan (Flu-Mel or FM) regimen represents another well-established RIC combination. Its development was led by the MD Anderson Cancer Center group during the early 2000s using melphalan dose of 100–180 mg/m2 [9]. Long-term follow up data of the Flu-Mel regimen demonstrated that escalating the melphalan dose to 140–180 mg/m^2^ for patients with active disease yielded survival outcomes similar to those achieved with 100 mg/m^2^ in patients transplant while in complete remission [8].

When compared to Flu-Bu, the reduced intensity Flu-Mel regimen has consistently demonstrated a significantly lower risk of relapse, particularly in patients with AML and MDS [26–29]. A recent comprehensive meta-analysis of 17 studies encompassing 10,396 patients further elucidates these dynamics, showing that while OS was often similar between the two, largely because the superiority of relapse protection in Flu-Mel is frequently offset by increased NRM, Flu-Mel provides a significant overall advantage in PFS. This survival benefit was especially pronounced in myeloid malignancies and elderly populations [30]. A propensity score analysis evaluating Flu-Mel at 100 mg/m^2^ vs. Flu-Mel 140 mg/m^2^ vs. Flu-Bu (target area under the curve 4000–6000 μmol/min per day), showed that the Flu-Mel 100 mg/m^2^ was associated with superior PFS and GVHD-free, relapse-free survival (GRFS) in a cohort of AML and MDS patients aged 60 years or older [31]. These findings suggest that Flu-Mel 100 mg/m^2^ may offer an optimal balance of efficacy and tolerability for older patients. However, the impact of the Flu-Mel regimen appears to be disease-specific. In contrast to the results seen in AML/MDS, meta-analytic data indicates that patients with lymphoma experience significantly worse OS and higher TRM with Flu-Mel compared to Flu-Bu [30]. This suggests that for lymphoid malignancies, the inherent toxicity of the Flu-Mel platform may negate its anti-tumor benefits, reinforcing the need to individualize RIC selection based on both patient age and the specific underlying disease.

To further enhance cytoreductive efficacy and intensity, fludarabine-based three-drug regimens have been developed, often incorporating a third agent such as thiotepa or low-dose TBI. A pivotal phase II clinical trial investigated this strategy using a platform consisting of fludarabine, melphalan (100–140 mg/m^2^), and either thiotepa (5 mg/kg) or 2 Gy TBI. This study evaluated high-risk patients receiving transplants from alternative donors – specifically haploidentical (Haplo) and 9/10 matched unrelated donor (MMUD) – utilizing post-transplant cyclophosphamide (PTCy) for GVHD prophylaxis. The results demonstrated the feasibility of this intensified approach, with durable donor chimerism achieved in 97–98% of evaluable patients and low 100-day NRM of 10% in the Haplo arm and 13% in the MMUD arm. At 1 year, PFS rates were 60% and 47% for Haplo and MMUD group, respectively. Notably, this study found that reducing the melphalan dose to 100 mg/m^2^ for older or more frail patients maintained comparable survival and disease control to the 140 mg/m^2^ dose [32].

Following these developments, the integration of TBI or thiotepa into Flu-Mel backbone has demonstrated promising results in other high risk populations, such as those with acute lymphoblastic leukemia (ALL) [33]. However, while melphalan-based RIC improves relapse protection compared to minimal-intensity regimens, the associated toxicity remains a concern for older and medically frail patients. To address this, a more recent phase II trial (NCT04191187) investigated an even lower melphalan dose of 70 mg/m2 (Flu-Mel70-TBI) in 34 patients undergoing haploidentical allo-HSCT with PTCy. By reducing the melphalan dose and moving it earlier in the conditioning sequence (day − 6), investigators aimed to minimize tissue injury and cytokine release syndrome (CRS) while maintaining anti-tumor efficacy. The results of this lower-dose approach were encouraging, meeting the primary endpoint with an 18-month DFS of 71% and OS of 73%. Despite the reduced intensity, the cumulative incidence of relapse at 18 months was remarkably low at 12% while the NRM was 18%. Furthermore, the regimen was associated with low rates of severe GVHD. These findings suggest that the Flu-Mel70-TBI platform offers a potent yet tolerable alternative for patients unfit for more intense conditioning, especially for the very old (> 70 years), effectively bridging the gap between minimal-intensity protocols and more toxic RIC variants [34].

RIC platforms incorporating low-dose TBI have also gained widespread use. This approach has yielded encouraging results in older or medically frail patients receiving HLA-identical sibling grafts for hematologic malignancies [35, 36] In a key study by Niederwieser et al. 52 elderly or medically unfit patients underwent allo-HSCT using a combination of fludarabine and 2 Gy TBI. Underscoring its clinical utility, this approach facilitated robust donor hematopoiesis with promising outcome. Durable donor chimerism was successfully attained in 88% of patients while 100-day regimen-related mortality remained low at 11%. OS at 1 year was reported at 44% [36].

To expand this minimal-intensity framework to the haploidentical setting, investigators integrated cyclophosphamide into the fludarabine/TBI backbone (Flu-Cy-TBI). The rationale for incorporating cyclophosphamide lies in its potent and versatile immunosuppressive properties; when administered pre-transplantation, it effectively suppresses the host immune system to prevent graft rejection, which is a significant barrier in HLA-mismatched transplants. Furthermore, this platform using high-dose PTCy to selectively deplete alloreactive T-cells while sparing non-alloreactive immunity. This dual application of cyclophosphamide allows for stable engraftment and the mitigation of severe GVHD without requiring intensive, toxic chemotherapy. The clinical utility of this Flu-Cy-TBI approach was demonstrated in a study of 68 patients conducted by Johns Hopkins and the Fred Hutchinson Cancer Center. Despite the high degree of HLA disparity among the donor-recipient pairs, the regimen maintained a favorable safety profile, with NRM of only 15% at 1 year and OS of 36% at 2 years [37].

To provide a broader perspective on the comparative efficacy of these diverse protocols, a large-scale national registry analysis by the CIBMTR recently evaluated the outcomes of 11,731 older patients (aged 50 years and above) with AML and MDS. This study, the largest of its kind to date, compared five commonly used fludarabine-based RIC regimens: Flu-Bu, Flu-Mel 100 mg/m^2^, Flu-Mel 140 mg/m^2^, Flu-Cy-TBI, and Flu-TBI. The analysis revealed significant differences in long-term survival and disease control among these platforms. A critical finding of this registry data was the trade-off between early toxicity and long-term disease control. While Flu-Mel regimens were associated with higher early NRM within the first six months, the late NRM was not significantly different between the groups. Moreover, both Flu-Mel 100 mg/m^2^ and Flu-Mel 140 mg/m^2^ demonstrated superior OS and DFS driven primarily by significantly lower relapse rates compared to all other regimens. Interesting, there was no significant difference in relapse rates or OS between the 2 melphalan-based regimens, suggesting that the 100 mg/m^2^ dose may be sufficient to provide robust anti-leukemic activity in older patients [38, 39], as previously suggested by our group [31].

The nuanced trade-offs between relapse control and regimen-related toxicity across these fludarabine-based platforms indicate that the “optimal” conditioning strategy is not universal, but rather highly dependent on the individual clinical context. This necessity for a tailored approach underscores the importance of the following sections, which will examine the critical factors in refining patient selection and the optimal of RIC protocols for specific hematologic malignancies.

### Optimizing RIC Candidacy: a Multidimensional Approach to Patient Fitness and Disease Biology

RIC regimens were initially developed to extend the eligibility of allo-HSCT to patients with advanced age or significant comorbidities who are unlikely to tolerate conventional MAC regimens. The selection of conditioning regimen intensity requires careful consideration of the balance between the risk of relapse and NRM, necessitating a collective review of patient characteristics, disease features, and transplant-related factors.

#### Patient-Related Factors: Age, Comorbidities, Performance Status, and Frailty

In recent years, several studies have demonstrated that RIC regimens can be safely applied in older patients (age ≥ 60 years) across different donor types [40–42]. Advanced age is one of the most important considerations, as older patients often have reduced physiologic reserve and increased vulnerability to toxicities. However, chronological age alone should not be the sole determinant in selecting the appropriate conditioning intensity. Assessment of comorbidities also plays a central role in patient selection [43]. A Hematopoietic Cell Transplantation–Comorbidity Index (HCT-CI) is a validated scoring system designed to assess pre-transplant organ dysfunction and predict the risk of NRM following allo-HSCT. Higher HCT-CI scores (score ≥ 3) are associated with increased transplant-related complications and inferior survival [44, 45]. Therefore, RIC regimens are often considered a more appropriate approach for these medically frail individuals [46–48].

Functional status, measured by Karnofsky Performance Status (KPS) and the Eastern cooperative oncology group (ECOG), also remains a critical prognostic marker. Data from the Acute Leukemia Working Party (ALWP) of EBMT involving nearly 3,000 AML patients showed that those with a KPS score ≤ 80% experienced lower NRM with RIC compared to MAC, without an increase in relapse rates [49].

While age, performance status and comorbidities provide foundational risk profiles, they often fail to capture the nuances of a patient’s physiologic “fitness”. Consequently, the assessment of frailty—a clinically recognizable syndrome characterized by multisystem deregulation and a decreased capacity to re-establish homeostasis after stress has emerged as a powerful, independent predictor of post-transplant mortality. A landmark study established and validated a novel HCT Frailty Scale designed for rapid clinical application [50]. This multidimensional tool incorporates eight variables; the Clinical Frailty Score (CFS), Instrumental Activities of Daily Living (IADL), grip strength, the Timed Up and Go Test (TUGT), self-rated health, history of falls, and biomarkers including albumin and C-reactive protein. This scale categorizes candidates into three distinct levels: fit, pre-frail, and frail. Notably, the study revealed that frailty is not exclusive to the elderly; one-third of patients classified as frail were between ages of 18 and 50, emphasizing that disease and prior treatments can accelerate physiological decline regardless of age. The clinical impact of frailty is profound; frail patients demonstrate significantly higher NRM and inferior OS. Importantly, frailty often exists independently of traditional markers; more than half of the frail patients in this study maintained a KPS of 90–100%, and nearly half had a low HCT-CI score [50, 51].

#### Disease-Related Factors: Disease Risk Index and Measurable Residual Disease

Beyond broad clinical indices, the specific disease risk and molecular status at the time of transplant are critical determinants in the selection of conditioning intensity. The Disease risk index (DRI), which integrates cytogenetic risk and remission status at transplant is well-established predictor of post-transplant survival [52]. According to CIBMTR data, patients with low or intermediate DRI achieved superior DFS and OS using MAC while survival was not significantly different in those with high- or very-high risk DRI transplanted using with MAC compared to RIC regimens, likely because the high inherent risk of relapse in these populations is not fully eliminated by increased intensity [53]. However, a more granular assessment of disease status, specifically the presence of measurable residual disease (MRD), provides greater clarity in choosing between RIC and MAC. Analysis from the BMT CTN 0901 trial demonstrated that next-generation sequencing (NGS)-positive AML patients receiving RIC had significantly higher relapse rates and inferior relapse-free survival and OS compared with those receiving MAC [54]. Research investigating the intensity of conditionings indicates that MAC is associated with a higher probability of early post-transplant MRD clearance [55]. These findings suggest that for patients with genomic evidence of MRD, the intensive cytoreduction provided by MAC may be essential to achieve early disease eradication, if they are younger and otherwise fit to receive MAC.

#### Integrated Risk Stratification Models

Recognizing that neither patient nor disease factors act in isolation, recent research has shifted toward composite risk models. While the HCT-CI focuses on patient fitness and the DRI focuses on disease aggressiveness, a new Hematopoietic Cell Transplant-Composite Risk (HCT-CR) model has been developed by our group. This model integrates both DRI and HCT-CI to categorize patients into four distinct risk groups with significantly different OS. This composite approach provides superior discriminative capacity compared to using either index alone, allowing clinicians to more accurately account for the interplay between a patient’s comorbidities and their disease biology [56, 57]. Further refinements have led to the development of an AML-specific Disease Risk Group (AML-DRG) and the AML-HCT-CR, which incorporated modern prognostic markers such as the ELN genetic risk and MRD status. In these models, detectable MRD before transplant is weighted heavily, as it was associated with a nearly two-fold increase in risk of death among patients in morphologic complete remission. By combining these molecular disease risks with patient-related factors (age, HCT-CI), the AML-HCT-CR model represents the most advanced tool for predicting survival in the modern era in patients with AML [58]. These comprehensive models not only better stratify prognosis but also will help guide therapy in the future in a more personalized approach.

### Comparative Efficacy of RIC Across Malignancies: From Randomized Trials to Real-World Evidence

While RIC regimens are utilized across a broad spectrum of hematologic malignancies, the evidence base for optimal conditioning intensity remains unevenly distributed. Prospective randomized trials have been primarily restricted to patients with AML and MDS. In contrast, the evidence in other hematologic malignancies relies on retrospective studies, which are often subject to inherent selection bias. This lack of randomized data is partly driven by the clinical difficulty of assigning intensity via a protocol rather than individualized risk. For example, clinicians are often reluctant to randomize young, fit patients with active disease to RIC due to high risk of relapse, or conversely, to assign frail patients to MAC, creating a significant barrier to conducting balanced, high-level comparative studies. Moreover, the lack of standardization across centers – including strong institutional preference and the vast array of available RIC and MAC protocols, further complicates the application of generalized trial results to specific clinical practices.

#### Acute Myeloid Leukemia (AML)/Myelodysplastic Syndrome (MDS)

Three prospective randomized trials have compared RIC and MAC regimens in patients with AML or MDS, most notably the BMT CTN 0901 trial. This prospective phase III randomized trial compared RIC protocols (Flu-Mel or Flu-Bu2) against MAC regimens (Flu-Bu4, Bu-Cy or Cy-12 Gy TBI) in HLA-matched transplant recipients, aged 18 to 65 years with an HCT-CI < 5. The trial was terminated early due to significantly higher incidence of relapse and worse survival in the RIC arm. While RIC initially demonstrated lower TRM, the primary endpoint—OS at 18 months was statistically comparable [59]. However, the long-term follow up result revealed that MAC provided superior OS compared to RIC (HR, 1.54; 95% CI, 1.07 to 2.20; P = 0.03). This superior advantage was driven by the fact that the excessive relapse rates in the RIC group ultimately outweighed its benefits in TRM protection [60].

A randomized phase III trial by the German AML group comparing a RIC regimen (Flu-8 Gy TBI) with a MAC regimen (Cy- 12 Gy TBI) in patients with AML, aged 18–60 years. Again, the trial was terminated early after enrolling 195 of the targeted 252 patients due to slow accrual rate [61]. With nearly 10 years of follow-up, the cumulative incidence of relapse was similar (30% in both groups). Despite a trend toward lower NRM (16% vs 26%), no significant difference was observed in DFS [62].

Similarly, the prospective phase III multicenter randomized RICMAC trial, conducted by the EBMT comparing a RIC regimen (Flu-Bu2) with a MAC regimen (Bu-Cy) in patients with MDS or secondary AML, age 18–65 years, showed comparable NRM, relapse, and survival [11, 63].

A recent prospective randomized trial (FIGARO study) compared a sequential transplant regimen consisting of fludarabine, amsacrine, cytarabine and busulfan (FLAMSA-Bu) with standard fludarabine-based regimens in patients with high-risk AML or MDS undergoing allo-HSCT from HLA-matched donors. The intensified RIC conditioning regimen (FLAMSA-Bu) did not improve transplant outcomes including OS, NRM or relapse rate, regardless of pretransplant MRD status [64]. Additionally, another study in previously untreated MDS patients with 5–19% marrow blasts found that a sequential transplant approach did not improve transplant outcomes compared with standard conditioning and was associated with a higher risk of chronic GVHD [65].

In addition, numerous retrospective multicenter studies have compared outcomes between RIC and MAC conditioning regimens in patients with AML or MDS undergoing allo-HSCT (Table 2). Most studies demonstrated that RIC is associated with a lower risk of NRM but a higher relapse, while overall survival and other transplant outcomes are generally comparable between the two groups [69]. The main limitation is that under RIC umbrella there is a variety of conditioning regimens associated with different transplant outcomes.

**Table 2:** Conditioning regimen intensity and transplant outcomes in patients with AML or MDS undergoing allogeneic hematopoietic stem cell transplantation

Clinical trial (Donor type)	Population (N)	Conditioning Regimens (N)		OS %	RFS/PFS/EFS %	NRM%	Relapse%	Acute GVHD grade II-IV%
		**MAC**	**RIC**					
**Prospective studies**								
Scott et al. [59, 60] 2017, 2021 (MSD, MUD)	272	Flu-Bu4 (87) Bu-Cy (40) Cy-12 Gy TBI (8)	Flu-Mel (21) Flu-Bu2 (89)	77.5 vs 68 (at 18 mo) (P < 0.01)	68 vs 47 (at 18 mo) (P < 0.01)	16 vs 4 (at 18 mo) (P = 0.002)	13.5 vs 48 (at 18 mo) (P < 0.01)	44.7 vs 31.6 (at 100 days) (P = 0.024)
				62 vs 49 (at 4 yr) (P = 0.022)	57.9 vs 33.7 (at 4 yr) (P < 0.0001)	25.1 vs 9.9 (at 4 yr) (P < 0.001)	19.8 vs 60.7 (at 4 yr) (P < 0.001)	N/A
Bornhauser et al. 2012 [61] (MSD, MUD, MMUD)	195	Cy-12 Gy TBI (96)	Flu-8 Gy TBI (99)	58 vs 61 (at 3 yr) (NS)	56 vs 58 (at 3 yr) (NS)	17 vs 13 (at 3 yr) (NS)	30 vs 30 (at 3 yr) (NS)	NS
Kroger et al. [11] 2017 (MSD, MUD, MMUD)	129	Bu-Cy (64)	Flu-Bu2 (65)	63 vs 76 (at 2 yr) (NS)	58 vs 62 (at 2 yr) (NS)	25 vs 17 (at 1 yr) (NS)	15 vs 17 (at 2 yr) (NS)	37.5 vs 32.3 (at 100 days) (NS)
**Retrospective studies**								
Luger et al. [66] 2012 (MSD, MUD, MMUD)	5179	5–8 Gy TBI with/without Cy Bu-Mel (3731)	2–5 Gy TBI Mel-others Low dose Bu-others (1041)	34 vs 33 (at 5 yr) (NS)	33 vs 30 (at 5 yr) (NS)	18 vs 12 (at 3 mo) (NS) At 3 yr: similar	32 vs 39 (at 5 yr) (NS)	45–47 vs 41 (at 100 days) (NS)
Martino et al. [67] 2013 (MSD)	878	Cy-TBI Bu-Cy (718)	Flu-alkylating agents (Bu, Mel, Cy, TT) Flu-2–4 Gy TBI (160)	56 vs 48 (at 7 yr) (NS)	54 vs 42 (at 7 yr) (P = 0.03)	23 vs 19 (at 7 yr) (NS)	23 vs 39 (at 7 yr) (P = 0.01)	10 vs 8 (at 100 days) (NS)
Damraj et al 2016 [27] (MSD, MUD)	134	Flu-Bu (47)	Flu-Mel (87)	53.1 vs 63.9 (at 2 yr) (NS)	48.7 vs 60.5 (at 2 yr) (P = 0.04)	15.7 vs 22.2 (at 2 yr) (NS)	35.6 vs 17.3 (at 2 yr) (P = 0.0058)	50.3 vs 45 (at 2 yr) (NS)
DI maggio et al. 2020 [68] (MSD, MUD, MMUD)	396	Flu-Bu 5300 (69)	Flu-Mel (246)	53.2 vs 56.7 (at 3 yr) (NS)	47.2 vs 47.4 (at 2 yr) (NS)	20.6 vs 31.7 (at 2 yr) (P = 0.049)	33.1 vs 19.4 (at 2 yr) (P = 0.024)	62.4 vs 56.1 (at 100 days) (NS)
Martino et al. [67] 2006 (MSD)	836 (MDS only)	Cy-8 Gy TBI Bu-Cy (621)	Flu-alkylating agents (Bu, Mel, Cy, TT) Flu-2–4 Gy TBI (215)	45 vs 41 (at 5 yr) (NS)	41 vs 33 (at 5 yr) (NS)	32 vs 22 (at 3 yr) (P = 0.04)	27 vs 45 (at 3 yr) (P < 0.01)	58 vs 43 (at 100 days) (P < 0.001)
**Sequential transplant**								
Craddock et al. [64] 2021 (MSD, MUD)	244	FLAMSA-Bu (122)	Flu-based (122)	60.9 vs 58.8 (at 2 yr) (NS)	54.2 vs 48.7 (at 2 yr) (NS)	20.5 vs 16.8 (at 1 yr) (NS)	26.7 vs 29.5 (at 2 yr) (NS)	8.3 vs 10.1 (at 100 days) (NS)

Legend: *Bu* busulfan, *Bu2* low-dose busulfan total 6.4 mg/kg for 2 days Bu4: full-dose busulfan total 12.8 mg/kg for 4 days, *Bu 5300* target AUC of busulfan 5300 μM min/day, *Cy* cyclophosphamide, *EFS* Event-free survival, *FLAMSA* Fludarabine, amsacrine, cytarabine, *Flu* fludarabine, *GVHD* graft-versus-host disease, *Gy* gray, *MAC* myeloablative conditioning regimen, *Mel* melphalan, *MMUD* mismatched-unrelated donor, *mo* month or months, *MSD* matched-sibling donor, *MUD* matched-urelated donor, *N/A* not available, *NRM* non-relapse mortality, *NS* not statistically significant, *OS* overall survival, *PFS* progression-free survival, *RIC* reduced-intensity conditioning regimen, *RFS* relapse-free survival, *TBI* total body irradiation, *TT* thiotepa, *yr* year or years

#### Acute Lymphoblastic Leukemia (ALL)

While TBI-based MAC is the standard of care for young and fit ALL patients due to its superior anti-leukemic effect compared to chemotherapy-based regimens, its use is limited by significant multi-organ toxicity. Consequently, for patients with advanced age or poor performance status, RIC regimens are employed. These protocols reduce the intensity of alkylating agents and TBI and incorporating fludarabine-based regimens to achieve sufficient lymphodepletion.

When comparing RIC and MAC in adults with Ph-negative ALL, a CIBMTR study found no significant differences in NRM or OS, even though the RIC patients were older. Notably, while survival outcomes were similar, the incidence of relapse was slightly elevated in the RIC cohort. In multivariable analysis, conditioning intensity was not independently associated with relapse risk, NRM, or OS [70]. Similarly, a recent EBMT retrospective study of 417 patients, aged > 45 years with ALL in first remission compared three commonly used RIC regimens: Flu-Bu, Flu-Mel, Flu-TBI. At 2 years, relapse incidence, leukemia-free survival, OS and NRM were similar across the three groups [71].

Several studies have demonstrated that an intensified RIC Flu-Mel based regimen by the addition of low-dose TBI or Thiotepa may improve transplant outcomes, reduce relapse rates, and prevent CNS relapse without increasing treatment-related mortality (Table 3) [33, 73–75]. These modifications may further enhance overall efficacy of RIC allo-HSCT in high-risk patients and need to be further studied prospectively.

**Table 3:** Conditioning regimen intensity and transplant outcomes in patients with ALL undergoing allogeneic hematopoietic stem cell transplantation

Clinical trial (Donor type)	Population (N)	Conditioning Regimens (N)		OS %	RFS/PFS/EFS %	NRM%	Relapse%	Acute GVHD grade II-IV%
		**MAC**	**RIC**					
Comparative retrospective study								
Marks et al 2010 [70] (MSD, MUD)	1521	Cy-TBI Bu-Cy (1428)	Flu-alkylating agents (Bu, Mel) Flu-TBI (93)	43 vs 38 (at 3 yr) (NS)	41 vs 32 (at 3 yr) (NS)	29 vs 26 (at 1 yr) (NS) 33 vs 32 (at 3 yr) (NS)	19 vs 26 (at 1 yr) (NS) 26 vs 35 (at 3 yr) (NS)	46 vs 39 (at 100 days) (NS)
Mothy et al. [72] 2020 (MSD)	576	Standard MAC (449)	Flu-alkylating agents (Bu, Mel, Cy) Flu- < 6 Gy TBI (127)	45 vs 48 (at 2 yr) (NS)	38 vs 32 (at 2 yr) (NS)	29 vs 21 (at 2 yr) (P = 0.03)	31 vs 47 (at 2 yr) (P < 0.001)	37 vs 39 (at 100 days) (NS)
Common RIC regimens								
Peric et al. [71] 2020 (MSD, MUD)	417		Flu-Bu (127) Flu-Mel (190) Ful-2 Gy TBI (100)	55 vs 50 vs 60 (at 2 yr) (NS)	43 vs 42 vs 45 (at 2 yr) (NS)	18 vs 22 vs 14 (at 2 yr) (NS)	40 vs 36 vs 41 (at 2 yr) (NS)	23 vs 27 vs 32 (at 100 days) (NS)
**Clinical trial** **(Donor type)**	**Population (N)**	**Conditioning Regimens (N)**		**OS %**	**RFS/PFS/EFS %**	**NRM%**	**Relapse%**	**Acute GVHD grade II-IV%**
Intensified RIC regimens								
Eder et al. [73] 2017 (MSD, MUD)	720	Cy-TBI (540)	Thiotepa-based regimens (180)	48 vs 46 (at 2 yr) (NS)	39 vs 33 (at 2 yr) (NS)	24.2 vs 23 (at 2 yr) (NS)	36 vs 43 (at 2 yr) (NS)	25.6 vs 22.6 (at 100 days) (NS)
Chen et al 2019 [74] (MSD, MUD)	317	Flu-Mel (162)	Flu-Mel(50) + TBI (61) Flu-Mel(75) + TBI (94)	45 vs 46 vs 45 (at 3 yr) (NS)	47 vs 53 vs 61 (at 3 yr) (NS)	10.5 vs 8.8 vs 8.7 (at 100 days) (NS)	14 vs 13.6 vs 6.8 (NS)	53 vs 52 vs 56 (at 100 days) (NS)
Battipaglia et al. [75] 2024 (MSD, MUD,MMUD, HAPLO)	265	Flu-Mel + low-dose TBI (117)	Flu-Mel + TT (148)	67 vs 56 (at 2 yr) (NS)	46 vs 57 (at 2 yr) (NS)	26 vs 20 (at 2 yr) (NS)	28 vs 23 (at 2 yr) (NS)	30 vs 30 (at 100 days) (NS)
Chumnumsiriwath et al. [33] 2024 (MSD, MUD,MMUD, HAPLO)	29	Flu-Mel + low-dose TBI Flu-Mel + TT		83 (at 2 yr)	83 (at 2 yr)	17.4 (at 2 yr)	0 (at 2 yr)	3.9 (at 100 days)

Legend: *Bu* busulfan, *Cy* cyclophosphamide, *EFS* event-free survival, *Flu* fludarabine, *GVHD* graft-versus-host disease, *Gy* Gray, *HAPLO* haploidentical donor, *MAC *myeloablative conditioning regimen, *Mel* melphalan, *MMUD* mismatched-unrelated donor, *mo* month or months, *MSD* matched-sibling donor, *MUD* matched-unrelated donor, *NRM* non-relapse mortality, *NS* not statistically significant, *OS* overall survival, *PFS* progression-free survival, *RIC* reduced-intensity conditioning regimen, *RFS* relapse-free survival, *TBI* total body irradiation, *TT* thiotepa, *yr* year or years

#### Lymphoma

RIC regimens are often preferred for patients with relapsed or refractory lymphoma because they allow exploitation of the graft-versus-tumor effect while maintaining an acceptable rate of NRM. (Table 4) A CIBMTR registry-based analysis of 396 patients with diffused large B-cel lymphoma (DLBCL) undergoing allo-HSCT compared outcomes after MAC (N = 165), RIC (N = 143) and NMA (N = 88) regimens. Not surprisingly, at 5 years, MAC was associated with higher NRM compared to RIC and NMA, whereas relapse/progression rates were lower with MAC. However, OS, PFS, and the incidence of both acute and chronic GVHD were similar among the three conditioning intensities [76]. In a large CIBMTR registry-based cohort study of 1,823 patients with non-Hodgkin lymphoma undergoing allo-HSCT with RIC/NMA regimens, outcomes were compared among Flu-Bu, Flu-Mel140, Flu-Cy, and Flu-Cy with 2 Gy TBI. At four years, OS varied across regimens, with the Flu-Mel140 cohort demonstrating inferior survival. Multivariable analysis identified that Flu-Mel140 was associated with higher NRM compared to Flu-Bu and an increased risk of chronic GVHD compared to Flu-Cy. Other regimens yielded comparable transplant outcomes [77]. These results highlight again, that Flu-Mel140 regimen is too intense for patients with lymphoma who are usually more heavily pretreated and often they have received an autologous stem cell transplant.

**Table 4:** Conditioning regimen intensity and transplant outcomes in patients with lymphoma undergoing allogeneic hematopoietic stem cell transplantation

Clinical trial (Donor type)	Population (N)	Conditioning Regimens (N)		OS %	RFS/PFS/EFS %	NRM%	Relapse%	Acute GVHD grade II-IV%
		**MAC**	**RIC/NMA**					
Non-Hodgkin lymphoma								
Bacher et al 2012 [76] (MSD, MUD, MMUD)	396	(165) Cy-TBI 55%	RIC (143) Flu-Bu 34% NMA (88) Flu-Cy 48%	21 vs 27 vs 29 (at 3 yr) (NS) 18 vs 20 vs 26 (at 5 yr) (NS)	19 vs 23 vs 27 (at 3 yr) (NS) 18 vs 15 vs 25 (at 5 yr) (NS)	55 vs 42 vs 34 (at 3 yr) (P = 0.003) 56 vs 47 vs 36 (at 5 yr) (P = 0.007)	23 vs 32 vs 37 (at 1 yr) (P = 0.043) 26 vs 35 vs 38 (at 3 yr) (P = 0.078)	43 vs 43 vs 44 (at 100 days) (NS)
Ghosh et al 2020 [77] (MSD, MUD)	1823		Flu-Bu (458) Flu- Cy-2 Gy TBI (89) Flu-Mel (885) Flu-Cy (391)	58 vs 67 vs 49 vs 63 (at 4 yr) (P < 0.001)	38 vs 51 vs 39 vs 35 (at 4 yr) (P = 0.07)	16 vs 17 vs 26 vs 17 (at 4 yr) (P < 0.001)	47 vs 33 vs 37 vs 50 (at 4 yr) (P < 0.001)	40 vs 29 vs 38 vs 34 (at 6 mo) (P = 0.12)
Nath et al. [78] 2024 (MSD, MUD)	279		RIC (110): Flu-Mel (55%) NMA (169): Flu-Cy-2 Gy TBI	Median OS: NR vs 103 mo (NS)	53 vs 61 (at 4 yr) (P = 0.045)	10 vs 1.8 (at 100 days) (NS)	N/A	48 vs 28 (at 6 mo) (P < 0.001)
Hodgkin lymphoma								
Genadieva-Stavrik et al. [79] 2016 (MSD, MUD)	312	BEAM BEAM-A Bu-Cy ± VP16 Cy-TBI (63)	Flu-Bu Flu-Cy Flu-Mel (249)	73 vs 62 (at 2 yr) (NS)	48 vs 36 (at 2 yr) (NS)	11 vs 12 (at 2 yr) (NS)	42 vs 52 (at 2 yr) (NS)	N/A
Ahmed et al. [80] 2020 (MSD, MUD)	492		Flu-Bu (102) Flu-Mel (318) Flu-Cy (72)	62 vs 59 vs 55 (at 4 yr) (NS)	29 vs 37 vs 25 (at 4 yr) (NS)	57 vs 47 vs 65 (at 4 yr) (P = 0.01)	10 vs 10 vs 3 (at 1 yr) (P = 0.02) 15 vs 17 vs 12 (at 4 yr) (NS)	46 vs 34 vs 26 (at 6 mo) (P = 0.02)

Legend: *Bu* busulfan, *Cy* cyclophosphamide, *EFS* event-free survival, *Flu* fludarabine, *GVHD* graft-versus-host disease, *Gy* Gray, *HAPLO* haploidentical donor, *MAC* myeloablative conditioning regimen, *Mel* melphalan, *MMUD* mismatched-unrelated donor, *mo* month or months, *MSD* matched-sibling donor, *MUD* matched-unrelated donor, *N/A* not available, *NRM* non-relapse mortality, *NMA* non-myeloablative conditioning regimen, *NS* not statistically significant, *OS* overall survival, *PFS* progression-free survival, *RIC* reduced-intensity conditioning regimen, *RFS* relapse-free survival, *TBI* total body irradiation, *TT* thiotepa, *yr* year or years

In the setting of Hodgkin lymphoma, an EBMT retrospective study evaluated outcomes for 312 patients with relapsed or refractory disease undergoing allo-HSCT with either MAC (n = 63) or RIC (n = 249). Following a median follow-up of 56 months, NRM was comparable between the two cohorts. While the MAC group demonstrated a trend toward reduced relapse rates and improved EFS, these differences did not reach statistical significance. Furthermore, OS at 24 months was similar between the MAC and RIC groups [79].

#### Myeloproliferative disorders (MPN)

Allo-HSCT remains the sole curative treatment for myelofibrosis (MF). However, the optimal conditioning regimens uncertain, particularly in older patients and those with significant co-morbidities. RIC regimens primarily Flu-Bu and Flu-Mel, are common employed to reduced treatment-related toxicity while maintaining adequate disease control. In retrospective European study involving 160 patients with MF, Flu-Bu was compared with Flu-Mel. Flu-Mel was associated with a lower relapse risk but higher rates of NRM and acute GVHD, with no significant OS between two regimens [81]. Similarly, another retrospective study involving 61 patients with MF compared Flu-Bu, Flu-Mel, and fludarabine/carmustine/melphalan (FBM). Although OS, relapse, and NRM were comparable across three regimens, Flu-Mel and FBM achieved higher rates of full donor chimerism at day + 30 and + 100 than Flu-Bu [82]. More recently, a large CIBMTR analysis evaluated 872 patients with MF, including 493 who underwent Allo-HSCT using RIC regimens (Flu-Bu and Flu-Mel). Among RIC recipients, Flu-Mel was associated with inferior OS (HR, 1.80; 95% CI, 1.15 to 2.18; P = 0.009), higher early NRM (HR, 1.81; 95% CI, 1.12–2.91; P = 0.01) and higher grade II-IV acute GVHD (HR, 1.45; 95% CI, 1.03 to 2.03; P = 0.03) compared with Flu-Bu [83]. These findings highlight the importance of balancing disease control and toxicity. Flu-Mel may offer improved disease control, it may also increase the risk of NRM and acute GVHD, where as Flu-Bu may provide favorable toxicity profile. However, prospective studies are warrant to determine the optimal conditioning regimens for MF.

### Conclusions and Future Direction

**Fig. 1:**
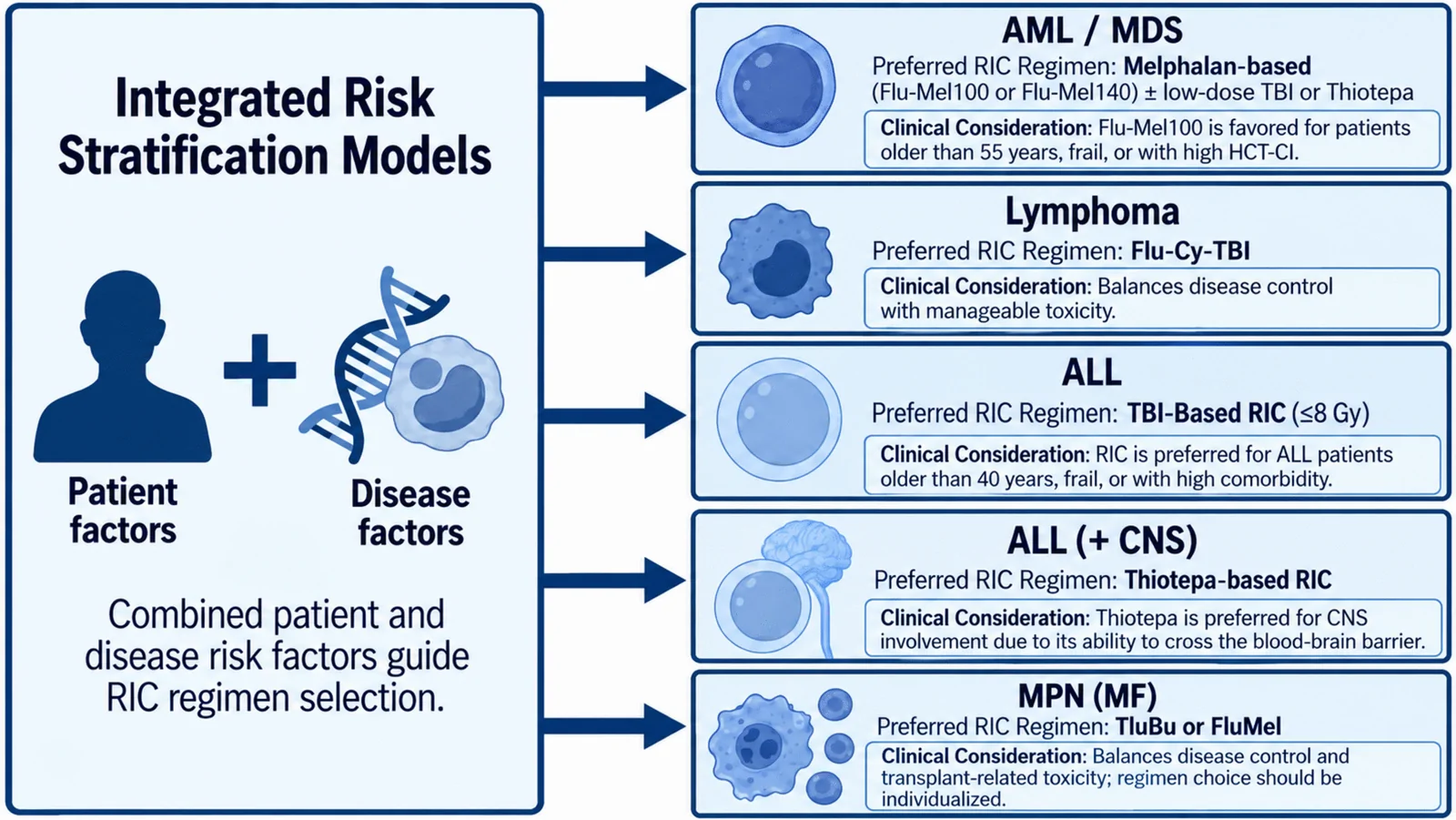
Individualized RIC Regimen Selection via Multi-Dimensional Risk Stratification

The evolution of Reduced-Intensity Conditioning (RIC) has fundamentally transformed the landscape of allogeneic hematopoietic stem cell transplantation (allo-HSCT), extending curative potential to older and medically frail populations who were previously ineligible for the procedure. By shifting the therapeutic focus from high-dose toxic cytoreduction to immune-mediated graft-versus-tumor (GVT) effects, RIC achieves survival outcomes comparable to Myeloablative Conditioning (MAC) across a diverse range of malignancies. However, achieving optimal outcomes necessitates a multidimensional selection process that balances patient-specific factors—such as comorbidities, performance status, and frailty—with disease parameters, including cytogenetic risk and MRD status. Reflecting this complexity, preferred RIC regimens vary significantly based on specific clinical scenarios (Fig. 1), ranging from very low-intensity protocols (e.g., Flu/2 Gy TBI) to those bordering on myeloablative intensity (e.g., Flu/Mel 140). This broad spectrum highlights the critical need to further sub-categorize these regimens and study them individually to better predict and improve transplant outcomes.

The future of RIC lies in highly personalized "precision conditioning." Refined dosing through real-time pharmacokinetic monitoring of fludarabine, busulfan, and melphalan offers the potential to minimize NRM while maximizing antitumor efficacy. Since relapse remains the primary barrier to long-term success, future strategies must integrate proactive post-transplant maintenance and novel immunotherapies, including bispecific antibodies. Finally, transitioning from retrospective data to large-scale prospective trials and standardized scoring systems will be essential to provide the high-level evidence needed for individualized clinical decision-making and improve transplant outcomes in the years to come.

## Data Availability

No datasets were generated or analysed during the current study.
